# Photon-Counting Chest CT at Radiography-Comparable Dose Levels: Impact on Opportunistic Visual and Semiautomated Coronary Calcium Quantification

**DOI:** 10.1097/RLI.0000000000001199

**Published:** 2025-04-25

**Authors:** Jonas Kroschke, Bjarne Kerber, Matthias Eberhard, Falko Ensle, Thomas Frauenfelder, Lisa Jungblut

**Affiliations:** Institute for Diagnostic and Interventional Radiology, University Hospital Zurich, University Zurich, Zurich, Switzerland

**Keywords:** photon-counting computed tomography, coronary artery calcification, calcium scoring, lung cancer screening, low-dose computed tomography

## Abstract

**Objectives:**

The introduction of photon-counting detector computed tomography (PCCT) has allowed for significant dose reductions compared to energy-integrating-detector CT, making it particularly relevant for applications such as lung cancer screening. Coronary artery calcification is an important incidental finding in lung cancer screening, warranting attention in this context. This study aims to assess the impact of dose reduction to levels comparable to that of a chest radiography on opportunistic evaluation of coronary artery calcification on PCCTs of the chest.

**Materials and Methods:**

Sixty-eight out of 115 patients with age >45 years and body mass index ≤30 kg/m^2^ undergoing noncontrast low- and chest-radiography-comparable-dose PCCT in the same session were included. Scans were performed at 100 kVp with image quality settings 12 (low-dose) and 2 (radiography-comparable-dose). Visual calcium scoring was conducted by 2 readers using 2 scoring approaches (CAD-RADS 2.0 and Shemesh). Semiautomated quantitative analysis was performed using commercially available software. Image quality was evaluated using 5-point Likert scales.

**Results:**

Sixty-eight patients (65.9 ± 8.6 years; 49 men) were subjected to evaluation. CTDI was lower for radiography-dose scans (0.11 mGy vs 0.68 mGy; *P* < 0.001). Image quality was found to be inferior for radiography-dose scans (4.01 vs 2.03; *P* < 0.001). In both visual scoring approaches, coronary calcification was scored significantly lower in radiography-dose scans (*P* < 0.001 for both) with almost perfect reader agreement (CAD-RADS score Cohen's kappa =0.82; Shemesh score Cohen's kappa =0.81), most importantly reclassification from mild to absent occurred for CAD-RADS score in 31%/21% of cases and for Shemesh score in 23%/15% of cases (reader 1/reader 2). Semiautomated assessment showed no significant differences between low and radiography dose (*P* = 0.121). Strong correlation between scores (Pearson's r = 0.98, *P* < 0.001) with good agreement (Cohen's kappa =0.61) was found.

**Conclusions:**

Coronary artery calcifications are underestimated on radiography-dose PCCT visually, whereas semiautomatic analysis provides more robust results. Visual underestimation of coronary artery calcification in low-dose imaging is further amplified with the additional dose reduction to radiography-comparable dose levels, indicating that while estimation of high cardiovascular risk is feasible, exclusion of such risk is not possible.

In recent years, lung cancer screening using low-dose computed tomography has been introduced and is still being introduced in many countries, as a result of the findings of large-scale studies, which have demonstrated a significant reduction in lung cancer mortality in at-risk populations.^1,2^ Risk factors for lung cancer overlap with cardiovascular risk factors, with cigarette smoking being a leading risk factor for both conditions. Consequently, coronary artery calcification (CAC) is a prevalent comorbidity that can be identified in screening CTs.^3,4^

Recommendations by leading clinical societies engaged in lung cancer screening have pointed out the need to report CAC, as it is linked to an unfavorable prognosis due to the potential for cardiovascular events and elevated mortality.^5,6^

Although electrocardiogram(ECG)-gated multidetector CT is regarded as the gold standard for CAC assessment, some studies have demonstrated the feasibility of CAC assessment in non-ECG-gated chest CTs, albeit with limitations regarding scan parameters.^7–10^ Recent guidelines have thus recommended different approaches for calcium quantification on non-ECG-gated chest CTs, including (semi)automated calcium scoring using the Agatston score and visual calcium scoring, which may employ ordinal scoring or visual assessment.^11,12^

The clinical introduction of photon-counting detector CT (PCCT) has enabled a further reduction in radiation dose to levels comparable to those of chest radiographs, while maintaining superior image quality compared to conventional CT systems at ultralow-dose levels.^13–16^ The single-layer semiconductor detector configuration enables PCCTs to directly quantify photon energy, thereby reducing electronic noise, enhancing spatial resolution, and facilitating the acquisition of spectral information.^17,18^ The assessment of CAC at radiography-comparable dose levels has not yet been assessed.

In the context of lung cancer screening, a further reduction in dosage is particularly relevant, given that participants are considered healthy at the time of their scan and that regular repetition of examinations results in an accumulation of radiation dose.

The aim of this study was to evaluate the impact of reducing radiation dose to chest radiography levels on the opportunistic assessment of CACs, using both quantitative and qualitative approaches in chest CT examinations performed with photon-counting CT.

## MATERIALS AND METHODS

### Patient Cohort

Ethics approval was obtained by the local ethics committee (KEK-ZH-NR. 2022-D0008) and the study was conducted in accordance with the principles of the Helsinki Declaration at a single site. Written informed consent was obtained from all patients prior to their participation in the study. The cohort was reported previously in a different research context.^19,20^

Prospectively, a total of 115 patients undergoing clinically indicated, non-contrast, non-ECG-gated, low-dose CT of the chest were enrolled between February and July 2023. Indications included lung cancer screening (n = 26), lung nodule work-up/follow-up (n = 22) and pulmonary infections (n = 14).

**FIGURE 1:**
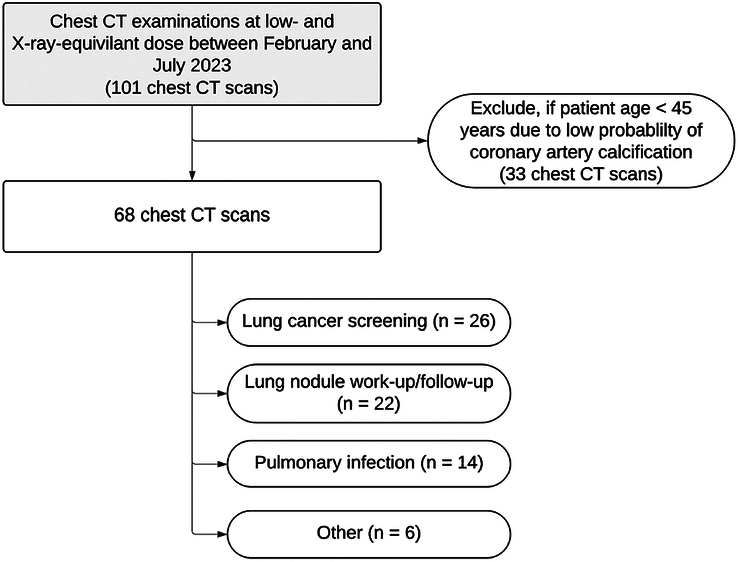
Patient flowchart.

Inclusion criteria were willingness to participate and age ≥18 years. For this study, patients were excluded, with age <45 years due to the low probability for CAC (n = 33)^21^ as well as patients with a body mass index (BMI) > 30 kg/m^2^ (n = 14). Patient selection is summarized in Figure 1.

### CT Examination Criteria

All examinations were conducted using a first-generation dual-source photon-counting detector CT scanner (NAEOTOM Alpha, Siemens Healthineers, Forchheim, Germany). The scanner is equipped with 2 cadmium-telluride photon-counting detectors (QuantaMax, Siemens Healthineers). All scans were performed in high-resolution mode (HR-mode) at 100 kVp with an image quality level of 12 as well as an image quality level of 2 (radiography-comparable-dose) and a pitch factor of 0.85. Tin filtration was used for all scans. The images were reconstructed in the axial plane using a medium-smooth soft tissue kernel (Br36). This kernel was chosen as it is designed to enhance the detection and quantification of high-contrast structures, specifically CACs, while minimizing noise. It helps mitigate beam-hardening and edge-enhancement artifacts that could interfere with quantitative assessments, particularly in low-dose settings. A standard slice thickness of 2 mm with an increment of 1 mm was selected. A quantum iterative reconstruction algorithm at standard strength (QIR 3) was applied as previously described.^19^

### Image Quality Assessment

Two independent readers (board-certified radiologists with over 10 years and 6 years of experience in cardiac imaging) evaluated the subjective image quality using a 5-point Likert scale with regard to overall image quality (1 = nondiagnostic, 2 = fair, 3 = moderate, 4 = good, 5 = excellent), sharpness of coronary arteries (1 = nondiagnostic reduction of sharpness, 2 = significantly reduced sharpness with blurring, 3 = reduced sharpness with blurring, 4 = minimally reduced sharpness, 5 = excellent sharpness), and image noise (1 = nonacceptable image noise, 2 = moderate noise, 3 = average image noise, 4 = fair image noise, 5 = minimal image noise).

### Visual CAC Assessment

Visual quantification of CACs was performed by the previously mentioned readers using 2 distinct ordinal scoring methods. Overall coronary calcification was evaluated in line with the method described by Cury et al in the CAD-RADS 2.0 recommendations, as this approach enables a comparison with the CAC score^22^ (Table 1). Furthermore, each coronary artery was rated separately, and the total score was calculated as the sum of each coronary artery as described by Shemesh et al^7^ (Table 1). Table 1 provides an overview of the 2 visual scoring approaches with corresponding CAC scores as described in the CAD-RADS 2.0 reporting recommendations.^22^

**TABLE 1:** Overview of the Visual Assessment Criteria for Subjective Calcium Scoring^7^ and Comparison With Agatston Scores as Suggested by CAD-RADS 2.0^22^

Agatston Score	Visual Assessment			
	Likert Score	Overall (CAD-RADS)	Individual Artery (Shemesh Score)	Overall (Shemesh Score)
-	0	-	Absent	Absent
1–100	1	Mild: 1–2 vessel with mild amount of plaque	Mild: <1/3 of length of coronary artery shows calcifications	Mild (1–3)
101–300	2	Moderate: 1–2 vessels with moderate amount; 3 vessels with mild amount of plaque	Moderate: 1/3–2/3 of length of artery shows calcifications	Moderate (4–7)
301–999	3	Severe: 3 vessels with moderate amount; 1 vessel with severe amount of plaque	Severe: >2/3 of length of artery shows calcifications	Severe (8–12)
>1000	4	Extensive: 2–3 vessels with severe amount of plaque	-	-

### Semiautomated Coronary Calcification Assessment

Semiautomated CAC quantification was performed by a radiologist in training (1 year of experience) using a commercially available cardiac analysis software (syngo.via, Siemens Healthineers) under the supervision of a board-certified radiologist (6 years of experience). Initially, automated calcium detection and classification to the corresponding coronary arteries were performed by the software for both low-dose and radiography-comparable-dose CT scans at the standard threshold of 130 HU. Because the 2-mm slice thickness used in this study deviates from the standard 3-mm slice thickness recommended for Agatston scoring, the resulting values are not directly comparable to the standardized Agatston score. However, as the reconstruction parameters remain consistent between low-dose and radiography-comparable-dose scans, the comparison within this study remains valid. Following this, results were validated by the reader and corrected if necessary. Corrections included the attribution of detected to calcifications to the correct coronary artery (mostly the decision between left main coronary artery and left anterior descending artery) as well as the exclusion of wrongly as CAC classified lesions (eg, calcifications of the aortic valve). Uncertain cases (eg, uncertain differentiation between left main coronary artery and left anterior descending artery) were discussed and consensus was reached. For comparison with visual scoring, CAC scores were stratified into groups of severity in accordance with CAD-RADS 2.0 reporting recommendations.^22^

### Statistical Analysis

The statistical analysis was conducted using the Python scipy-library version 1.13.1.^23^ Unless otherwise indicated, variables are reported as mean ± standard deviation. For group comparison of semiautomated and visual CAC scoring results nonparametric Wilcoxon signed-rank testing was employed. Pearson correlation was used to assess linear relationships between continuous variables in semiautomated CAC scoring. Interobserver agreement as well as agreement between quantitative and qualitative assessment was assessed using linearly weighted Cohen's κ statistic. к-results were classified qualitatively by score according to Landis and Koch^24^ (slight agreement, 0.01–0.20; fair agreement, 0.21–0.40; moderate agreement, 0.41–0.60; substantial agreement, 0.61–0.80; almost perfect agreement, 0.81–0.99). A *P* value of less than 0.05 was considered to indicate statistical significance.

## RESULTS

### Cohort

Of 115 prospectively examined patients, 33 patients were excluded as they were younger than 45 years with a low risk for CAC and 14 due to a BMI >30 kg/m^2^ (Fig. 1). The final cohort of 68 patients (49 men) had a mean age of 65.9 ± 8.6 years. A summary of patient characteristics is provided in Table 2.

**TABLE 2:** Summary of Patient Characteristics

Patient Demographics and Characteristics			
	Total	Male	Female
	n = 68	n = 49	n = 19
Age (SD)	65.9 (8.6)	65.0 (8.1)	68.2 (8.9)
BMI (SD)	24.2 (3.3)	25.2 (2.8)	21.8 (3.2)

BMI, body mass index.

### Comparison of Imaging Parameters and Dose

For low-dose CT and radiography-comparable-dose CT tube current was modulated depending on patient size with a mean tube current of 79.4 ± 18.9 mAs for low-dose and 13.3 ± 3.1 mAs for radiography-comparable-dose at a fixed tube voltage of 100 kVp. This resulted in a mean CTDIvol of 0.68 ± 0.16 mGy for low-dose and a significantly lower CTDIvol of 0.11 ± 0.03 mGy for radiography-comparable-dose (*P* < 0.001). Details on the imaging parameters used in this study are described in Table 3.

**TABLE 3:** Details of the Imaging Parameters Used in This Study

Parameter	Parameters in This Study	
	Low-dose CT	Radiography-comparable-dose CT
ECG gating	None	
Filter	Tin filter	
Tube voltage	Fixed: 100 kVp	
Tube current	Low tube current, modulated based on patient size:	
	79.4 ± 18.88 mAs	13.28 ± 3.09 mAs
Scan mode	Low pitch spiral (pitch factor 0.85)	
Slice thickness/increment	2.0 mm/1.0 mm	
Collimation	Single collimation width: 0.2 mm Total collimation width: 24 mm	
Revolution time	0.5 s	
Field-of-view	Variable to cover full chest	
Reconstruction algorithm	Quantum iterative reconstruction	
Kernel	Medium-smooth soft tissue kernel (Br36)	
Dose	CTDIvol:	
	0.68 ± 0.16 mGy	0.11 ± 0.03 mGy

CT, computed tomography. ECG, electrocardiogram.

### Subjective Image Quality Assessment

**FIGURE 2:**
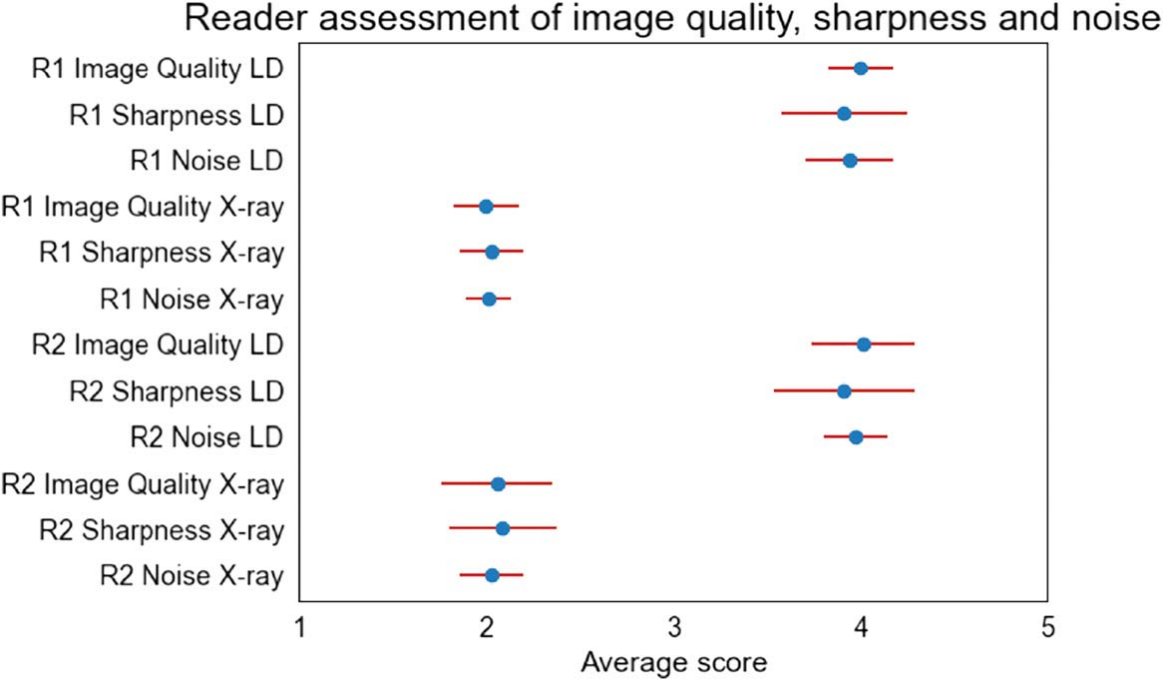
Subjective image quality assessment (mean ± standard deviation) by reader 1 (R1) and reader 2 (R2) for low-dose CT (LD) and radiography-comparable-dose CT (RD) in terms of overall image quality (1 = nondiagnostic, 2 = fair, 3 = moderate, 4 = good, 5 = excellent), sharpness (1 = nondiagnostic reduction of sharpness, 2 = significantly reduced sharpness with blurring, 3 = reduced sharpness with blurring, 4 = minimally reduced sharpness, 5 = excellent sharpness) and image noise (1 = nonacceptable image noise, 2 = moderate noise, 3 = average image noise, 4 = fair image noise, 5 = minimal image noise). For both readers image quality and sharpness were scored significantly lower with higher image noise for radiography-comparable dose CT.

Both readers assessed image quality in terms of overall image quality, sharpness and noise. Low-dose examinations were rated as good for overall image quality with minimally reduced sharpness and fair image noise by both readers (Fig. 2). Radiography-comparable-dose CT examinations were rated lower in terms of overall image quality (*P* < 0.001), with most assessments falling into the range of “fair.” Image sharpness was also found to be significantly reduced (*P* < 0.001), accompanied by noticeable blurring and moderate noise (Fig. 2). One radiography-comparable-dose examination was deemed nondiagnostic concerning overall image quality by 1 reader. Accordingly, image noise was rated “fair” on average for low-dose, while being rated significantly higher for radiography dose CT (*P* < 0.001). Interrater agreement was found to be almost perfect for all assessment categories (weighted Cohen's kappa values of 0.87 for image sharpness, 0.95 for noise, 0.94 for image quality).

### Subjective Image CAC Assessment

#### Overall Subjective CAC Assessment (CAD-RADS)

**FIGURE 3:**
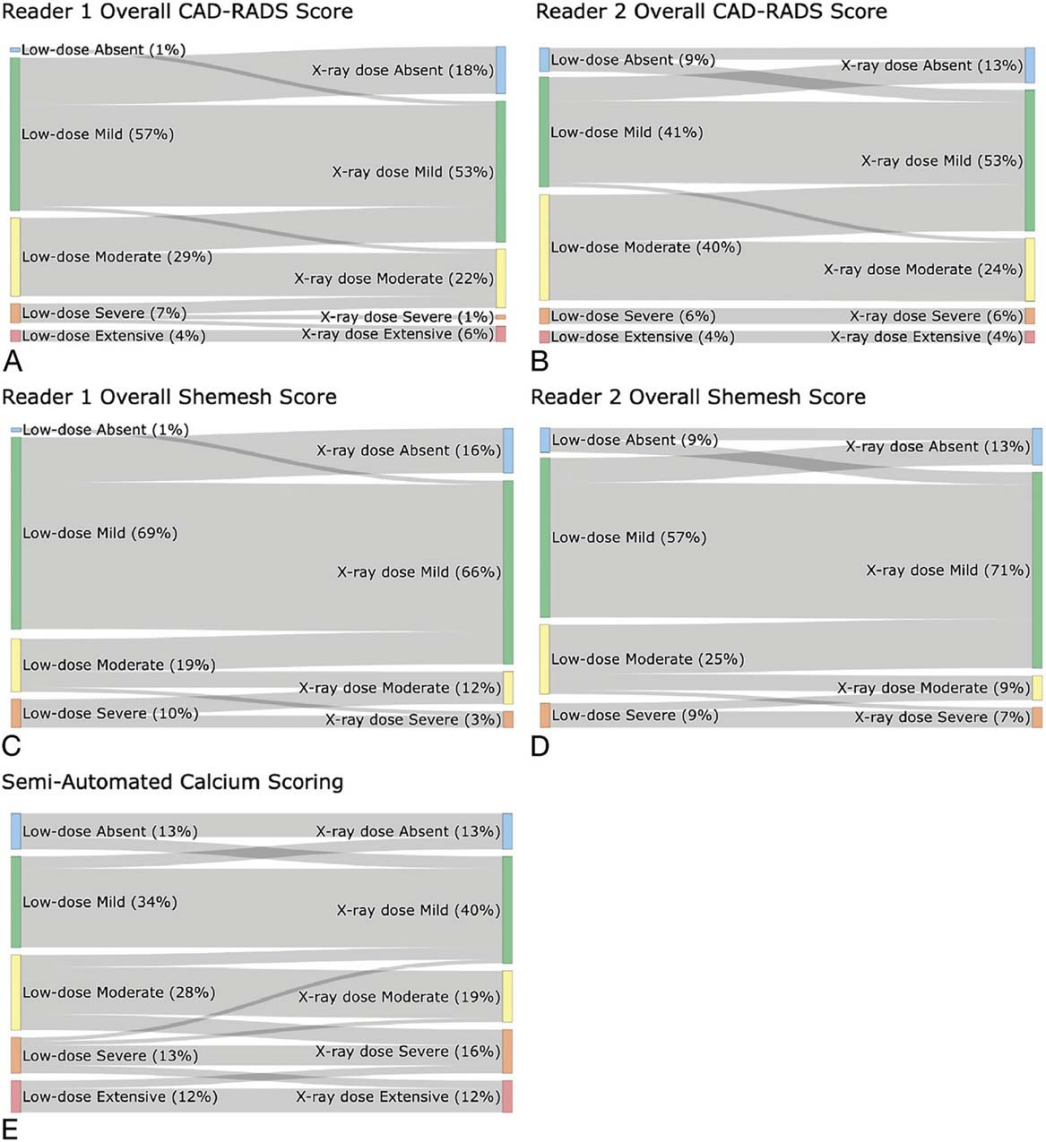
Sankey-Plots showing changes in classification from low-dose to radiography-dose CT for visual scoring using the CAD-RADS method for overall scoring (A and B) and the overall Shemesh score (C and D), as well as semiautomated analysis (E).

Overall subjective CAC assessment according to criteria defined in CAD-RADS 2.0, exhibited a significant difference between dose levels (reader 1: *P* < 0.001; reader 2: *P* = 0.003). Both readers demonstrated a tendency to reclassify overall calcifications into a lower severity group (Figs. 3A, B). For instance, reader 1 reclassified 12 examinations (31%) and reader 2 reclassified 6 examinations (21%) from mild CAC in low-dose imaging to absent in radiography-dose imaging. Likewise, reader 1 reclassified 9 examinations (45%) and reader 2 reclassified 12 examinations (44%) from moderate to mild. Interrater agreement for the overall subjective assessment was nearly perfect (weighted Cohen's kappa = 0.82). A minority of exams was “upgraded” into a higher group, most notably 1 examination from severe to extensive by reader 1.

#### Subjective CAC Assessment by Each Coronary (Shemesh Score)

**FIGURE 4:**
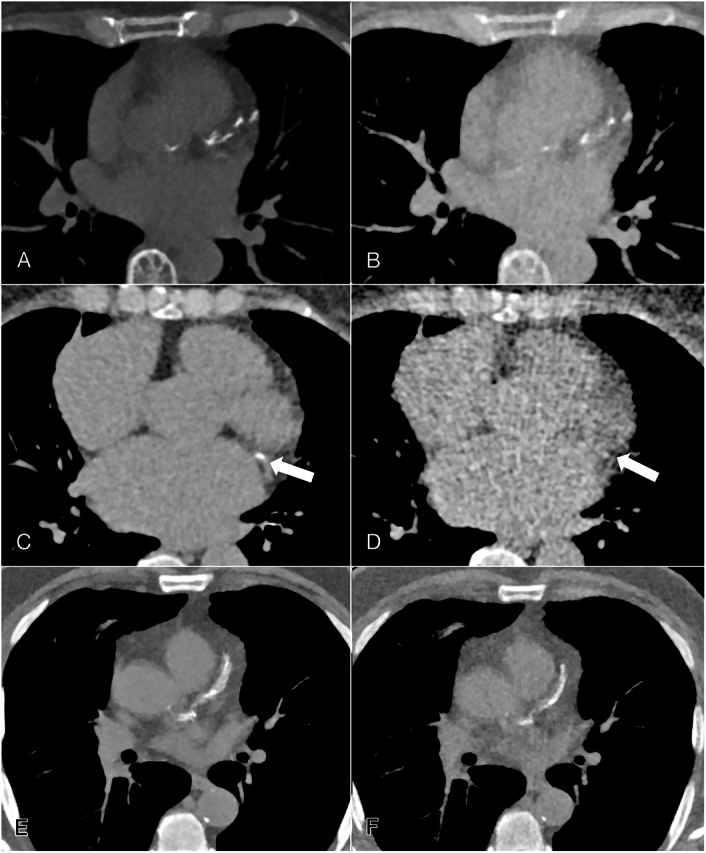
Examples of coronary calcifications at low-dose (A, C, E) and radiography dose (B, D, F) in axial plane in noncontrast, non-ECG-gated PCCTs of the chest. First row shows coronary calcifications that were scored as moderate by both readers at low-dose (A) and radiography dose (B) in a 67-year-old female. Second row shows calcification (arrow) scored as mild at low-dose (C) by both readers with the calcification not being picked up at radiography dose (D), thus being scored as absent by both readers in a 76-year-old female. Third row shows calcifications scored as severe at low-dose (E), while scored extensive at radiography dose (F) by 1 reader in a 68-year-old male.

Each coronary artery was individually assessed visually and a total score calculated as the sum of individual scores, as proposed by Shemesh et al.^7^ Significant differences in ratings were found between low-dose and radiography dose in RCA (reader 1: *P* = 0.004; reader 2: *P* = 0.003), LAD (reader 1 *P* = 0.007, reader 2 *P* = 0.008), and LCX (reader 1: *P* = 0.005 and reader 2: *P* = 0.002). No significant differences were observed in the LM (reader 1: *P* = 0.122; reader: 2: *P* = 0.307), with only a small number of patients exhibiting calcifications. Both readers reclassified patients primarily into the next lower category, for example, 11 examinations (23%) by reader 1 and 6 examinations (15%) by reader 2 from mild to absent from low to radiography dose or 8 examinations (62%) by reader 1 and 12 examinations (71%) by reader 2 from moderate to mild (Figs. 3C, D). Interrater agreement was almost perfect for RCA and LAD (weighted Cohens kappa = 0.90 and 0.85, respectively) and substantial for LM and LCX (weighted Cohens kappa = 0.69 and 0.78, respectively). Global comparison of Shemesh scores shows substantial agreement between readers (weighted Cohens kappa = 0.81). A minority of exams was “upgraded” into a higher group, most notably 1 examination each from moderate to severe by both readers. Figure 4 provides some examples of differences.

### Semiautomated Coronary Calcium Quantification and Scoring

**FIGURE 5:**
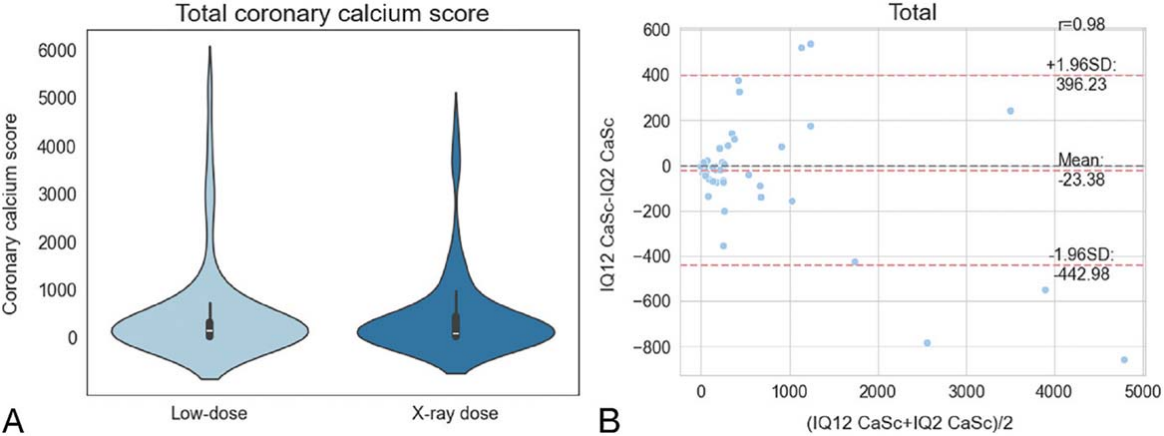
A, Violin plot showing similar distribution of coronary artery calcification scores determined by semiautomated quantification for low-dose and radiography-comparable-dose CTs. B, Bland-Altman plot of the CAC scores determined by semiautomated assessment between low-dose and radiography-dose scans for both readers showing only limited bias with random scatter and most data points falling within limits of agreements with some outliers showing higher variability. High correlation was observed between dose levels (Pearson's r = 0.98).

Data indicate a slight decline in Agatston scores form low-dose (mean: 490.28 ± 1002.25) to radiography-dose examinations (mean: 442.0 ± 867.76); however, not significantly (*P* = 0.121). High correlation was observed between dose levels (Pearson's r = 0.98) (Fig. 5A). The Bland-Altman plot reveals only limited bias (mean = −23.38) with random scatter and most data points falling within limits of agreement (Fig. 5B). Some outliers can be observed, especially for larger scores, indicating slightly higher variability for these cases, suggesting that differences may increase at higher calcium burden levels. Conversely, data points that formally fall within the limits of agreement but demonstrate relevant differences between low- and radiography-comparable dose may still indicate clinically relevant variability for individual subjects. Overall substantial agreement was found for CAC scores between dose levels (weighted Cohen's kappa = 0.61). Most reclassifications from low- to radiography-comparable-dose were “downgrades,” with 3 examinations reclassified from mild to absent (13%), 3 from moderate to mild (16%), and 2 each from severe to moderate (22%) and extensive to severe (25%). Several reclassifications took place into a higher group, for example, 4 examinations from moderate to severe (21%) and 2 examinations from severe to extensive (22%).

Looking at coronaries individually, only in the LCX significant differences between scores were observed (*P* = 0.016) with moderate correlation and moderate agreement between dose levels (Pearson r = 0.61, *P* < 0.001; weighted Cohens kappa: 0.44). All other coronaries showed no significant differences in calcium score with excellent correlation between dose levels (LM: *P* = 0.989, Pearson r = 0.87, *P* < 0.001; LAD: *P* = 0.081, Pearson r = 0.94, *P* < 0.001; RCA: *P* = 0.404, Pearson r = 0.94, *P* < 0.001). Agreement for individual coronaries ranged from moderate to substantial (weighted Cohens kappa: LM = 0.54; LAD = 0.58; RCA = 0.69).

Detailed examination of semiautomated classification (Fig. 3E) revealed some reclassifications did occur from low to radiography dose, albeit at a lower rate compared to subjective scoring. Specifically, 3 examinations were reclassified from mild to no calcification, and 3 from moderate to mild calcification. In contrast to the subjective assessment, a greater number of examinations were reclassified to a higher group. Specifically, 3 examinations were reclassified from absent to mild, 4 from moderate to severe, and 2 from severe to extensive.

### Comparison of Semiautomated and Visual Scoring

**FIGURE 6:**
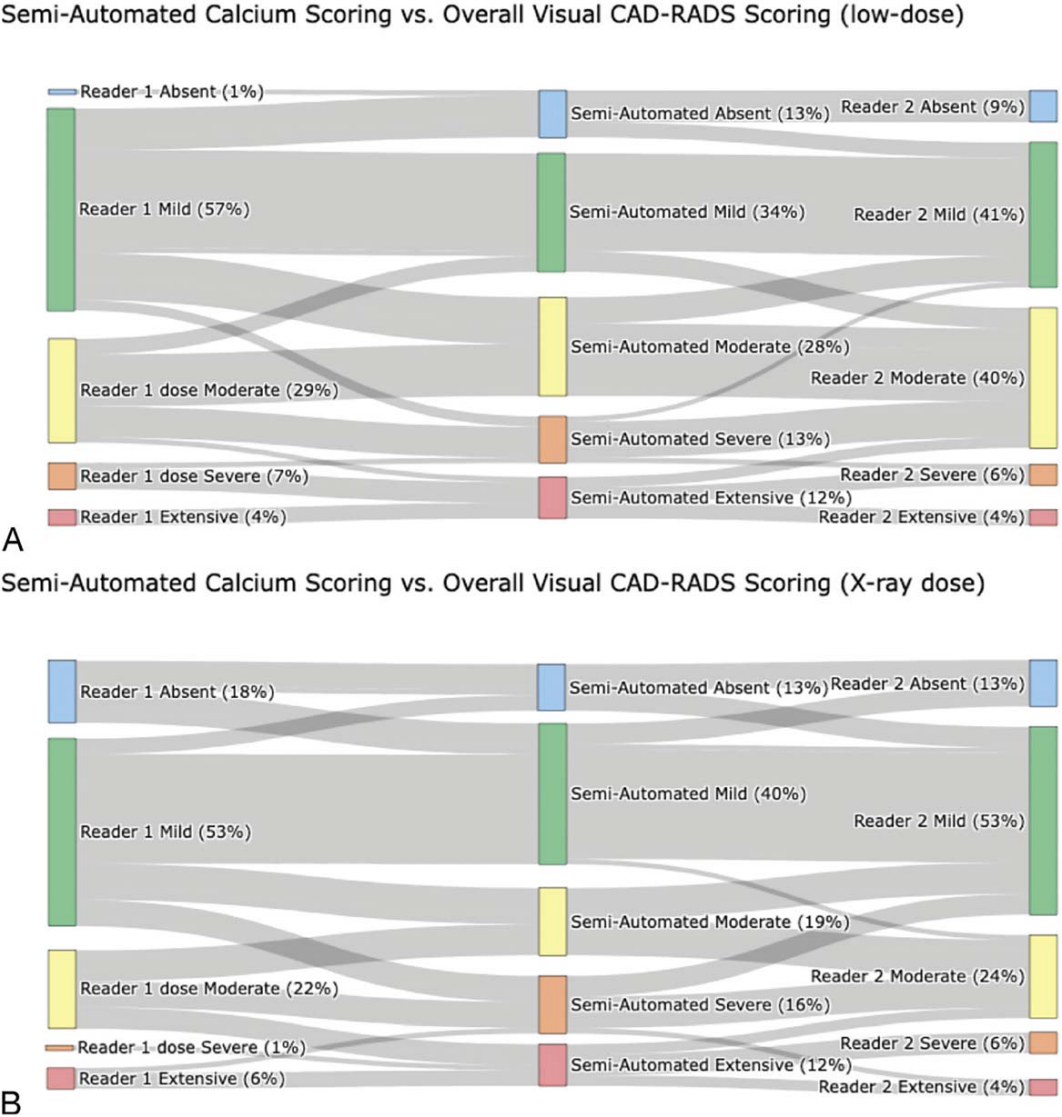
Sankey plots showing the differences in the Coronary Artery Calcification classification between semiautomated scoring and visual scoring at low-dose (A) and radiography-comparable dose (B). Agreement between readers and semiautomated assessment was found to be moderate to substantial for low dose and moderate for radiography dose.

Comparison of semiautomated and visual scoring shows differences in the classification of patients at both low dose and radiography dose (Fig. 6). At low-dose agreement between semiautomated calcium, scoring was found to be moderate compared to reader 1 (weighted Cohen's kappa = 0.52) and substantial compared to reader 2 (0.64). While at radiography-dose agreement between semiautomated calcium scoring was found to be moderate compared to both readers (weighted Cohen's kappa reader 1 = 0.51, reader 2 = 0.52).

## DISCUSSION

The objective of this study was to assess the impact of PCCT of the chest at dose levels comparable to those of a chest radiography on opportunistic semiautomated scoring using the Agatston score and visual coronary calcium quantification as further dose reduction is of particular interest in many settings. For example, in lung cancer screening, strict dose management is relevant, most importantly due to the potential population-wide public health impact resulting from the risk of cancer induced by exposure of participants to ionizing radiation. Based on data from the National Lung Screening Trial, it has been estimated that 1 radiation-induced cancer could occur in every 2500 participants,^25^ thereby underscoring the necessity for further dose reduction.

Subjective assessment confirmed the obvious reduction in image quality, sharpness of delineation of coronary arteries, and increase in image noise that is associated with a 6-fold reduction of radiation dose from low- to radiography-comparable dose. However, both readers determined that CT examinations at radiography-comparable dose were sufficient to allow for a visual scoring of CAC of individual coronaries, except for 1 reader's assessment of 1 examination, confirming visual scoring is indeed possible at radiography-comparable dose levels. Feasibility of dose reduction to radiography-comparable dose levels has already been shown for the quantification of pulmonary emphysema.^19^

The impact of dose reduction on visual calcium scoring was found to be statistically significant, resulting in a notable reclassification of calcium scores to lower severity categories. This was observed in both CAD-RADS-like overall scoring and even more pronounced in the ordinal scoring approach described by Shemesh et al.^7^ Severity of CAC is an important predictor of patient outcome in cardiovascular disease^27,28^ and a lower classification can therefore have a direct clinical impact. Especially the false reclassification of patients as having no CAC may be problematic, as the absence of CAC is regarded as a relevant predictor of long-term cardiovascular disease-free survival.^28^

Semiautomatic scoring demonstrated greater resilience to dose reduction, with a relatively limited influence on calcium scores. Although no significant overall differences were identified, individual examinations exhibited reclassification, albeit to a lesser extent than visual scoring. For instance, 13% of examinations were reclassified from mild to absent using semiautomated scoring, compared to a range of 15%–31% using visual scoring. The comparison of the classification of semiautomated calcium scoring compared to visual scoring revealed some differences with mostly moderate agreement between the 2 approaches. Previous studies have shown similar results with a limited comparability of quantitative and qualitative calcium scoring in the low-dose setting,^29,30^ which this study confirms for radiography-comparable-dose CT.

The findings of this study align with those of previous studies. Shemesh et al in their ground laying work first described the scoring methods used in this study, showing the relationship between the rate of cardiovascular deaths and an increasing CAC score.^7^ Similarly, Budoff et al demonstrated excellent correlations between ECG-gated and ungated low-dose CT.^31^ More recently, meta-analyses conducted by Kim et al^9^ and Osborne-Grinter et al^10^ further confirmed agreement between CAC scores in gated and nongated low-dose chest CT studies. However, some studies have indicated that dose reduction may result in an underestimation of coronary calcification. Xia et al demonstrated in a direct comparison of ECG-triggered cardiac CT versus nontriggered low-dose chest CT that although there is almost perfect agreement between CAC detection, low-dose CT mainly underestimates CAC scores, resulting in inaccurate risk categorization for patients with a BMI ≥ 30.^32^ Similar results were obtained by Allio et al in their study of 1511 patients receiving standard- and low-dose CT with systematic underestimation for low-dose CTs.^33^ Some authors suggest that the underestimation might be caused by a lower signal-to-noise ratio due to dose reduction, which can lead to smaller calcifications being missed.^33^ For semiautomated calcium scoring, lowering the tube voltage influences density measurements as Hounsfield units are standardized at a tube voltage of 120 kVp.^34^ Additionally, more lower energy photons are absorbed at lower tube voltages.^34^ Various factors can influence the assessment of CACs in radiography-comparable dose PCCT, and some approaches could enhance the results presented in this study. While the Br36 kernel, used in this study, is designed to enhance the detection and quantification of CAC, Yang et al demonstrated that although Br36 provides a higher contrast-to-noise ratio, kernels such as Bv40 and Bv36 may offer advantages for calcium scoring, at least in the context of standard cardiac PCCT.^35^ Additionally, adjusting the iterative reconstruction strength could impact the calcium scoring results. Although increasing the reconstruction strength would aggressively reduce image noise, potentially improving the visibility of low-contrast structures, it may not be as beneficial for high-contrast structures like CAC. This is because higher reconstruction strengths could lead to oversmoothing, increased partial volume artifacts, or altered Hounsfield unit values, all of which could negatively affect both visual and semiautomated CAC assessment.

This study has some limitations. The comparison of low-dose CT with radiography-dose CT is lacking a comparison with the recommended clinical standard for coronary calcium scoring as pointed out in Table 1. Some study participants did not undergo lung cancer screening CT examinations, resulting in a different risk profile for cardiovascular disease. Due to the limited number of patients in this cohort, the impact of patient BMI on calcium scoring in low-dose chest CT was not considered. Moreover, patients with a BMI > 30 kg/m^2^ were excluded introducing a selection bias. Previous studies have suggested that BMI can influence the results of calcium scoring in low-dose chest CT.^32^ In the present study, patients with a BMI > 30 kg/m^2^ were initially excluded from the analysis in order to ensure the creation of a more homogeneous dataset and to minimize confounding factors such as increased image noise and beam-hardening effects, which could introduce additional variability. Further studies should include a larger patient cohort, thus allowing for an evaluation of the impact of patient BMI. Future studies should specifically investigate whether radiography-comparable dose PCCT is feasible in this patient group. The findings of this study suggest that the limitations of the radiography-comparable dose approach may be amplified in obese patients, potentially making examinations at these dose levels impractical for this population.

The results of our study suggest that additional dose reduction to levels similar to chest radiography exaggerates underestimation of CAC, particularly in the context of opportunistic visual calcium scoring. It is important to consider the underestimation when interpreting CAC results on low-dose and even radiography-comparable-dose CTs as exclusion of cardiovascular disease cannot confidently be made when no coronary calcification is visible as small plaques might not be picked up due to the changes in density and/or the higher amount of image noise. The impact on semiautomated calcium scoring was less pronounced, indicating that the implementation of semiautomated scoring in ultralow-dose settings may prove advantageous in comparison to visual scoring. However, as there was only moderate agreement between quantitative and qualitative assessment, it remains unclear, which approach should be favored in terms of cardiovascular outcome, warranting further research.

## ORCID ID

*Jonas Kroschke https://orcid.org/0000-0003-1225-3016*
